# Quartz-bearing rhyolitic melts in the Earth’s mantle

**DOI:** 10.1038/s41467-022-35382-3

**Published:** 2022-12-15

**Authors:** Luigi Dallai, Gianluca Bianchini, Riccardo Avanzinelli, Etienne Deloule, Claudio Natali, Mario Gaeta, Andrea Cavallo, Sandro Conticelli

**Affiliations:** 1 Dipartimento Scienze della Terra, Sapienza—Università di Roma, P.le A. Moro, 5, 00185 Roma, Italy; 2 INGV, Via di Vigna Murata 605, 00143 Roma, Italy; 3 CNR—IGG, Area della Ricerca di Pisa, Via Moruzzi, 1, 56127 Pisa, Italy; 4 Dipartimento di Fisica e Scienze della Terra, Università di Ferrara, Via G. Saragat, 1, 44122 Ferrara, Italy; 5 Dipartimento di Scienze della Terra, Università di Firenze, Via G. La Pira, 4, 50121 Firenze, Italy; 6 CRPG, UMR 7358 CNRS-Université de Lorraine, 15 rue Notre Dame des Pauvres, 54501 Vandoeuvre les Nancy, France; 7 CNR—IGAG, Area della Ricerca di Roma-1, SP 35d, 9, 00010 Montelibretti RM, Italy; 8 Certema S.c.a.r.l., S.P. del Cipressino km 10, 58044 Borgo Santa Rita, Cinigiano GR Italy

**Keywords:** Solid Earth sciences, Petrology, Geochemistry

## Abstract

The occurrence of rhyolite melts in the mantle has been predicted by high pressure-high temperature experiments but never observed in nature. Here we report natural quartz-bearing rhyolitic melt inclusions and interstitial glass within peridotite xenoliths. The oxygen isotope composition of quartz crystals shows the unequivocal continental crustal derivation of these melts, which approximate the minimum composition in the quartz-albite-orthoclase system. Thermodynamic modelling suggests rhyolite was originated from partial melting of near-anhydrous garnet-bearing metapelites at temperatures ~1000 °C and interacted with peridotite at pressure ~1 GPa. Reaction of rhyolite with olivine converted lherzolite rocks into orthopyroxene-domains and orthopyroxene + plagioclase veins. The recognition of rhyolitic melts in the mantle provides direct evidence for element cycling through earth’s reservoirs, accommodated by dehydration and melting of crustal material, brought into the mantle by subduction, chemically modifying the mantle source, and ultimately returning to surface by arc magmatism.

## Abstract (web summary)

The paper reports the occurrence of quartz-bearing rhyolitic melt inclusions and interstitial glasses within peridotite xenoliths. Their O-isotope composition proves crustal derivation and cycling into the mantle at convergent plate margins.

## Introduction

Felsic melts, erupted as quartz-bearing rhyolite in volcanic environment or stagnating within the crust to generate granitic rocks, are thought to be originated within the shallow continental crust, either via partial melting of continental crustal rocks or by extensive differentiation of basaltic melts, at temperatures lower than 800 °C^1–3^.

Several experimental studies demonstrated that, at convergent plate margins, dacite-rhyolite melts can also be generated at higher temperature (>1000 °C) and pressure (>2 GPa) by partial melting of continental crustal lithologies either during sediment subduction^4,5^ or through exhumation of subducted continental crust^6^. Despite being postulated experimentally^3^, direct evidence for the occurrence of rhyolite melts at mantle depths has never been found.

Experimental petrology indicated that rhyolitic melts formed into the mantle from partial melting of subducted crustal material react with the peridotite to form metasomatised mantle domains^7–10^ at convergent plate margins, alternatively or in concomitance with fluids from dehydration of the oceanic slab. This process may produce a veined metasomatic mantle^11^ or a melange^12^ that may undergo melting at different degrees and generate potassic (HK-calc-alkalic to shoshonitic) and ultrapotassic magmas at destructive plate margins^13,14^.

Composite (i.e., veined) mantle-derived ultramafic xenoliths are rare in post-collisional tectonic settings.

One of the best suites of these type of xenoliths is from Cabezo Negro de Tallante, in South-East Spain^15–18^, where they were erupted 2 Ma, along with Na-alkaline basaltic ejecta of a monogenetic volcanic centre.

The area is characterized by a complex geodynamic evolution resulting from the Tertiary closure of the westernmost sector of the Tethys Ocean and subsequent continental collision between Africa and Eurasia plates^19^. Such evolution involved subduction, slab rollback, continental collision and eventually extensional episodes, and was characterized by calc-alkaline to ultrapotassic subduction-related magmatism (12–6 Ma), followed by the aforementioned Na-alkaline products^20^ (Supplementary Fig. 1). Accordingly, the ultramafic mantle xenoliths erupted at Tallante likely represent portions of a supra-subduction mantle wedge^15–18^.

The “Tallante” composite xenoliths are found among a wide population of regular type peridotites, and display extreme heterogeneity^15–18^. They are characterized by reactive felsic veins, mainly made of orthopyroxene, plagioclase and quartz, separated from the host peridotite by an orthopyroxene-rich reaction zone^21,22^. Previous studies on the same composite xenoliths analyzed in this study showed variable elemental and isotopic composition (Sr, Nd, Pb, and O). Mineral phases (plagioclase and orthopyroxene) within the vein recorded typically crustal values, whilst those from reaction zone and the surrounding peridotite showed progressively less extreme isotope compositions^21,22^. These features suggest that the mantle xenoliths derived from the interaction of mantle peridotite with crustal melts, rather than adakitic ones, as previously inferred^15–17^. The persistence of marked isotopic heterogeneities and the lack of re-equilibration between the different domains also suggest that metasomatic process mantle occurred shortly before the xenolith exhumation^21,22^.

In two of these xenoliths, we found rare intergranular high-silica melt ± quartz inclusions within orthopyroxene crystals of the peridotite, confirming the metasomatic nature of these Si-rich melts in the supra-subduction mantle realm. This data are processed using petrological modelling to constrain pressure and temperature of the trapped rhyolitic melt inclusion and hosted quartz, and to constrain the interaction between the crustal-derived rhyolites and the surrounding peridotite mantle. Quartz crystals in the glass inclusions within orthopyroxene, and/or crystallized from the interstitial rhyolitic melts within the felsic vein, were measured in situ for their O-isotope compositions.

## Results and discussion

### Petrographic and textural characteristics

**Fig. 1:**
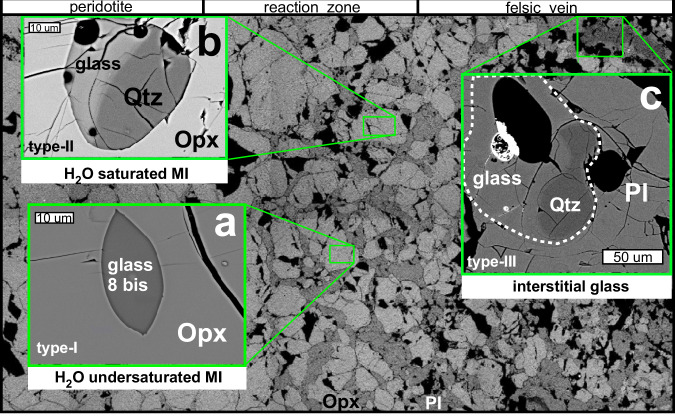
Texture of rhyolite inclusions. SEM back scattered electron (BSE) images showing the textures of rhyolite glasses (**a**–**c**) found in the different portions of the composite xenolith TL112 (large photo).

The TL112a and TL112b are quite unique composite samples among a large set of metasomatized mantle xenolith^18^, from Tallante area (SE Spain). They are characterized by a peridotite portion, and a reaction zone consisting of large orthopyroxene with equigranular, polygonal texture, transitioning into a felsic vein where the orthopyroxene is intergrown with plagioclase (Fig. 1).

As the amount of infilling plagioclase increases, the orthopyroxene crystal size decreases and the crystals shapes become less defined with curved contacts. Overall, the textural evidence of the vein suggests that orthopyroxene appears early in the sequence, being followed by orthopyroxene + plagioclase and quartz (Fig. 1). Equilibration P-T conditions of the newly formed parageneses were estimated in the range of 850–1050 °C and 0.7–0.9 GPa^18^.

Three texturally and compositionally distinct types of rhyolite glasses were found in the different portions of the sample, from the surrounding peridotite to the vein passing through the reaction zone. The large orthopyroxene crystals, characterizing the reaction zone, contain micrometric melt inclusions (20–30 μm), named hereafter Type-I, that show net rounded borders with host orthopyroxene (Fig. 1a). They can be interpreted in terms of droplets of silica-rich rhyolitic melts trapped into orthopyroxene crystals formed at the expenses of mantle olivine. Type-I inclusions show homogenous aspect with no vesicles (gas/liquid bubbles). High SEM magnification reveals that the melt inclusion edges are characterized by cuspate offshoots reflecting overpressure conditions of the trapped melt in the orthopyroxene (Fig. 1a). Accordingly, overpressure of melt inclusions appears to be the cause of orthopyroxene crystal lattice decrepitation, as testified by the development of secondary fractures in the host mineral. This is clear evidence for the entrapment of the melt into the orthopyroxene at higher pressure, likely in excess of 1 GPa.

A second type of glass inclusions, named hereafter Type-II (Fig. 1b), are also hosted into orthopyroxene of the reaction zone. They have greater dimensions (up to 80 μm) than the former, contain bubbles indicating water-saturated melt composition, and are characterized by the notable presence of daughter quartz crystals, that crystallized from the melt inclusions (Supplementary Table 1).

The last type of glasses, named hereafter Type-III, is represented by large melt films, blebs, and interstitial silica-oversaturated glasses found in the inner part of the felsic vein (Fig. 1c). They have irregular shapes and are interstitial between big poikilitic plagioclase crystals. These glassy films and pockets reach 100 μm in diameter, and often surround anhedral quartz crystals, crystallized within the felsic veins.

### Composition of rhyolite glasses

**Fig. 2:**
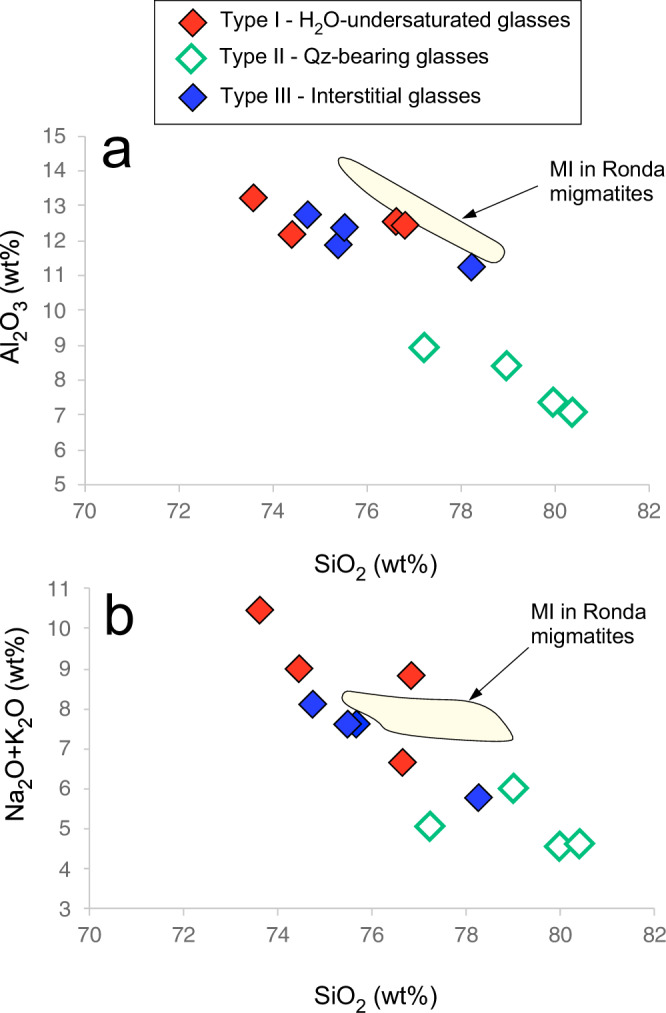
Composition of rhyolite glasses. SiO_2_ vs. Al_2_O_3_ (**a**) and vs. alkali (Na_2_O + K_2_O) (**b**) covariation diagrams for the three glass types recorded in the TL112a and TL112b composite xenoliths from Tallante, South-East Spain. Compositions of melt inclusions (MI) from Ronda migmatites are also reported^46^. Abbreviations: Qz = quartz.

All the analyzed glasses have rhyolitic compositions (Supplementary Table 1), sometimes extremely rich in SiO_2_ (>80 wt. %) and poor in CaO and TiO_2_ (0–1.13 wt.%, and 0–0.54 wt.%, respectively). SiO_2_ vs. Al_2_O_3_ and SiO_2_ vs. alkali covariation diagrams (Fig. 2), suggest differentiation occurred from the most silica poor Type-I inclusions to the most silica-rich Type-II melts. The extreme SiO_2_ enrichments could be also related to quartz dissolution during decompression^23,24^.

Type-I glasses, devoid of bubbles and daughter minerals, are expression of H_2_O-undersaturated melts (H_2_O contents between 0 and 1 wt. %; Supplementary Tables 1 and 4) and show a relatively large spread in SiO_2_ (73.6–76.9 wt.%). Type-II (Qz-bearing) glasses are comparatively enriched in SiO_2_, MgO and FeO and depleted in Al_2_O_3_ and alkalis with respect to Type-I ones and have water contents as high as 10 wt.% (%; Supplementary Tables 1 and 4). Type-III interstitial glasses have silica contents (SiO_2_ = 75.4–78.3 wt.%) within the range of Type-I inclusions, with only one datapoint at higher values, close to Type-II ones. The glasses described here, although occurring in a mantle xenolith, are compositionally similar to glass inclusions observed in high-grade metamorphic rocks that underwent anatectic processes^25^, and particularly, to migmatite rocks of Ronda, in the Betic Cordillera^26^.

### Oxygen isotope composition of quartz

In situ oxygen isotope measurements (Supplementary Table 2; Supplementary Fig. 1) were performed by SIMS on micrometric quartz within Type-II and -III glasses. SIMS analyses were performed on quartz minerals only, because instrument mass fractionation related to matrix effects in high-SiO_2_ glass may result into inaccurate measurements^27^. Based on quartz-glass fractionation at elevated temperatures (>500 °C), the δ^18^O values [δ^18^O = ^18^O/^16^O_sample_/^18^O/^16^O_std_ − 1)*1000] of melt inclusion should be enriched relative to the coexisting quartz by 0.3 to 0.6‰^28^.

Oxygen isotope geochemistry has been proven resolutive to distinguish crustal vs. mantle origin of mineral phases^29,30^. While unmodified mantle rocks are characterized by a tight δ^18^O variation (4.6–5.6‰)^31^, crustal lithologies show a wide oxygen compositional range, due both to the interaction with the hydrosphere and to temperature dependant O-isotope fractionation. This results in rocks with low (high-T fractionation) or high (low-T fractionation) δ^18^O values. Although the interpretation of low δ^18^O values (close to mantle ones) may be uncertain, high δ^18^O values are undoubtedly distinctive of continental crust. Several lines of evidence have already suggested that the peridotitic mantle at Tallante interacted with crustal material^21,22^. Our new in situ O-isotope measurements show that δ^18^O of quartz trapped within the rhyolitic glass (δ^18^O_qz_) is high and variable (8.3–14.1‰). Such a heavy oxygen isotope composition unequivocally proves that the rhyolitic glass and coexisting quartz cannot be produced by differentiation of mantle rocks, thus confirming the crustal origin of the rhyolitic melts from which they crystallized. On the other hand, the large variations in δ^18^O_qz_ may result from: (i) different proportions of felsic melt and host peridotite during crust-mantle interaction; (ii) diffusion-assisted re-equilibration with the surrounding phases of the peridotite mantle parageneses^32^.

**Fig. 3:**
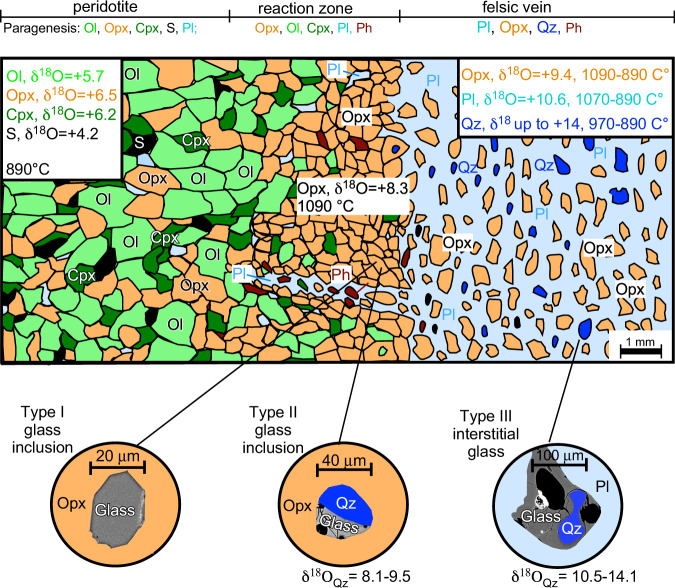
Mineralogy and isotopic variations along the xenolith. Sketch reporting the mineralogical variation observed in the composite xenolith TL112 and the related oxygen isotopic values (expressed as δ^18^O ‰, with respect to the SMOW standard). The observed variation allows to schematize within the xenolith three distinct textural domains: peridotite, reaction zone, felsic vein. Emphasis is given to the textural framework in which distinct types of glass inclusions are observed in orthopyroxene crystals of the reaction zone, and to the glassy films recorded around quartz within the vein. Equilibration temperatures inferred by the MELTS modelling are also reported. Abbreviations: Ol olivine; Opx orthopyroxene; Cpx clinopyroxene; S spinel; Pl plagioclase; Ph phlogopite; Qz quartz.

The highest values (13.4–14.1‰) were measured in large anhedral quartz (>200 μm) crystals enveloped by rhyolitic glass (Type-III) and plagioclase within the inner portion of the plagioclase-rich vein (Fig. 3), while the lowest ones (8.3–9.5‰) belong to subhedral micrometric (20–40 μm) crystals in Type-II glass inclusions within the orthopyroxene of the reaction zone (Fig. 3). Quartz crystals within the vein are variable in δ^18^O_qz_ values (10.5–12.5‰; Supplementary Table 2), which are collectively averaged at 11.5‰ to calculate a possible quartz-plagioclase equilibrium fractionation temperature of 845 °C (δ^18^O_pl_ = 10.5‰ in the vein)^21,33^.

The low δ^18^O_qz_ values of micrometric quartz enclosed in type-II glass inclusions (Supplementary Table 2) argue for significant isotopic diffusion-assisted re-equilibration. At mantle temperature, oxygen self-diffusion rates in quartz are 4–5 orders of magnitude faster than in orthopyroxene^32^, thus its oxygen isotopic composition was completely re-equilibrated with that of the large volume of the host orthopyroxene. At the same time these crystals are enclosed in a portion of the xenolith (the Reaction Zone) and their relatively low δ^18^O values may also be originally derived from a melt that had already reacted with the peridotite attaining slightly lower oxygen isotopic composition (Fig. 3).

Differently, large quartz crystals associated to Type-III glasses in the vein show higher and more variable δ^18^O_qz_ values, from 10.5 to 14.1 ‰ (Table S2), the latter being the highest δ^18^O values ever recorded in pristine mantle rock worldwide. These high values were measured in crystals from the inner portion of the vein where they likely underwent little, if any, retrograde re-equilibration or interaction with the surrounding peridotite. These quartz crystals may have partially re-equilibrated only with the surrounding plagioclase crystals, which have themselves relatively high (although not as high as quartz) δ^18^O values (Fig. 3). In general, the lack of complete isotopic re-equilibration between the different phases (including quartz) occurring in this veined mantle xenoliths testifies that the metasomatic processes occurring in this section of the lithospheric mantle were still active when the xenoliths were brought to the surface by alkaline magmas.

### Genesis of the rhyolite melts

Type-I glasses are slightly-modified pristine felsic metasomatic melts that veined the mantle, as also suggested by their textural features. Thermodynamic modelling using MELTS^34–36^ at *P* = 1 GPa and H_2_O = 1 wt.% conditions, predicts that the H_2_O-undersaturated compositions of Type-I melt inclusions are characterized by high liquidus temperatures (*T* = 1280–1060 °C) and co-saturation with garnet, implying a derivation from partial melting of high grade felsic metamorphic rocks. The occurrence of residual garnet in the protolith of the felsic metasomatic melts was also suggested by Avanzinelli and co-authors^22^ on the basis of REE pattern of clinopyroxene from the metasomatized peridotite matrix of the same xenolith. In addition, the lack of Ti also indicates the occurrence of residual rutile during crustal melting.

**Fig. 4:**
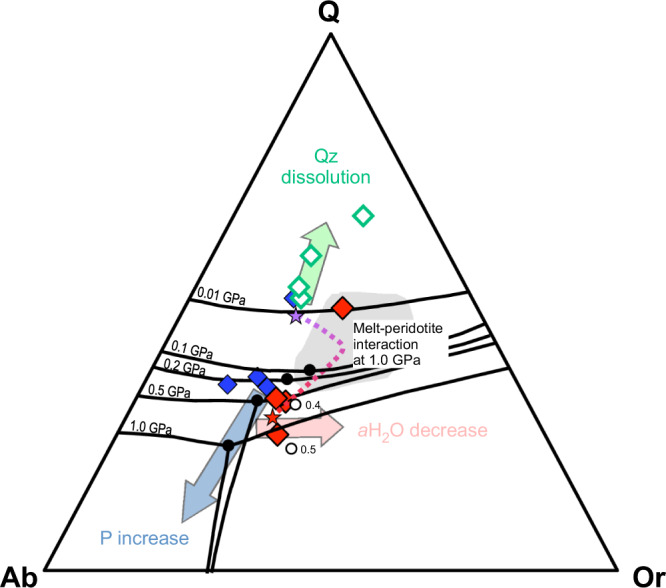
Ternary quartz-albite-orthoclase diagram illustrating normative compositions of melt inclusions and interstitial glasses (corrected for normative anorthite after^4^). Triangle apex are: Q quartz; Ab albite; Or orthoclase. The composition of the rhyolite melt-peridotite interaction trend modelled by MELTS (stars and dotted line) and the field of rhyolite melt inclusions in Ronda peridotite^46^ are shown for comparison. Eutectic and minimum points of the sub-aluminous haplogranite system at aH_2_O = 1.0 (black dots), 0.5 and 0.4 (white dots) are also reported (adapted from the literature^38,60^). Abbreviations: Qz quartz.

The composition of the rhyolite melts slightly deviates from that of the eutectic in the haplogranitic system^37,38^ (Fig. 4). The evolution of the measured glasses is rather complex since it may be affected by two different processes such as (i) polybaric evolution and (ii) interaction of the pristine rhyolitic melts with the surrounding peridotite.

As evident from the composition of Type-I glasses, the pristine crustal-derived rhyolitic melt was extremely Ca-poor, therefore not initially saturated with plagioclase. The occurrence of abundant plagioclase in the felsic vein requires the interaction with the surrounding peridotite. The petrographic and textural features of the reaction zone and the felsic vein indicate that the rhyolitic melts reacted with the peridotite country rock with destabilization of olivine, clinopyroxene and spinel and with neo-formation of orthopyroxene and plagioclase.

Model calculations by MELTS (Supplementary Table 3) predict that Type-I melt interacting with peridotite at 1 GPa may evolve by crystallizing orthopyroxene and subsequently orthopyroxene + feldspar + quartz. According to the model, quartz starts to crystallize cotectically with orthopyroxene at 970 °C and, subsequently, with decreasing temperature at 890 °C reacts with the olivine contributing to orthopyroxene formation. The thermodynamic model perfectly reproduces the mineralogical assemblage of the different textural domains of the studied xenolith. This is possible, however, only if the rhyolite melt is at significantly higher T than surrounding peridotite (*T*_rhyolite_ ≥ 1130 °C, *T*_peridotite_ = 890 °C; Table S3). Indeed, quartz crystallization is prevented when assuming a higher temperature for the peridotitic mantle.

In Fig. 4, the measured glasses are plotted in the Qz-Ab-Or diagram, together with the MELTS model (Supplementary Table 3) and the experimental cotectic curves at decreasing pressures^37,38^. (1–0.1 GPa). The figure shows that both polybaric evolution and interaction with the peridotite can drive the composition of the rhyolite melt towards higher silica contents. The same process may be responsible for the increase in water contents.

The silica-richest type-II glasses plot at too high silica for both processes, in agreement with the hypothesis of quartz dissolution during decompression. Few type-III glasses plot slightly outside the modelled interaction trend, suggesting that their chemistry is dominated by polybaric evolution. This is consistent with the textural position of type-III glasses (in the innermost portion of the vein) and their high δ^18^O values, both arguing for little, if any, interaction with peridotite (Fig. 3).

**Fig. 5:**
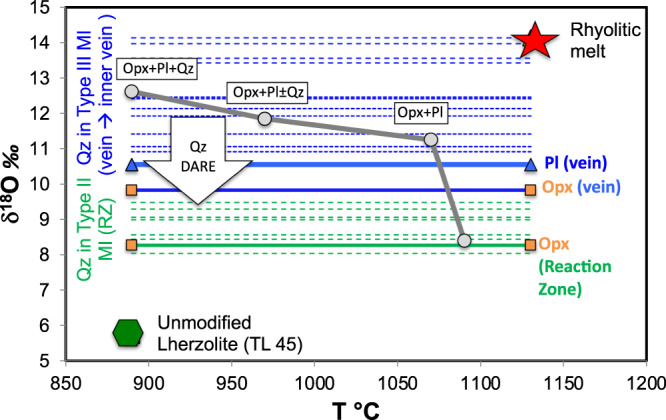
Variation of δ^18^O with temperature during the interactions between the rhyolitic melts and the peridotite mantle. Grey circles and lines represent the theoretical variation of δ^18^O values recalculated by mass balance from the MELTS model (Supplementary Table 3). Horizontal lines represent the δ^18^O values measured in the quartz crystals (this study) and mineral separates^21^ of the different portions of the composite xenolith (orange = reaction zone; blue = vein). The mineralogy predicted by the MELTS model are also reported. Abbreviations: Opx orthopyroxene; Pl plagioclase; Qz quartz; RZ reaction zone; MI melt inclusions. The vertical arrow represents the effect on δ^18^O of diffusion assisted re-equilibration of quartz crystals with surrounding Opx in the reaction zone (within type II Melt Inclusions).

In Fig. 5 we calculated the hypothetical oxygen isotope values during the different steps of the metasomatic reactions predicted by the thermodynamic model, starting from typical mantle values for the peridotites (Supplementary Table 3) and felsic melts with δ^18^O = 14 ‰. As the reaction proceeds, the relative proportions between felsic melt and peridotite increases. This results in oxygen isotope compositions that become progressively heavier towards the innermost portion of the vein, where plagioclase becomes dominant and quartz eventually crystallizes. The model is able to reproduce the isotopic variation observed in the mineral separates from the different portions of the xenolith^21^, as well as some of the δ^18^O values measured in the quartz crystals. Most likely, quartz crystal having highest δ^18^O values belong to domains formed during the latest stages of felsic melts injection into peridotitic mantle, where they only interacted with previously reacted domains (i.e., already with high δ^18^O value), and crystallized quartz with almost pristine crustal signature.

### Subduction of continental crust and mantle metasomatism

**Fig. 6:**
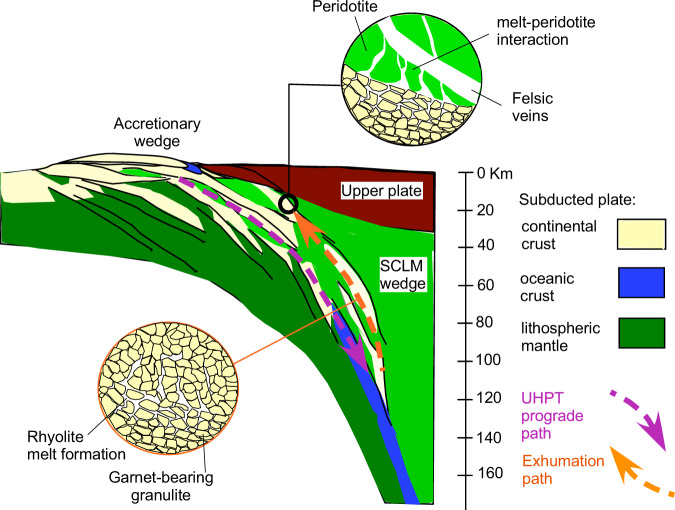
Cartoon showing the subduction and exhumation of continental crust slivers with the formation of rhyolite melts. Note that in the conceived model the subducted crustal blocks (and the entrained rhyolite melts) are subsequently exhumed to shallower mantle depths during slab roll back processes in relation to their relative buoyancy. Finally, rhyolite melts segregate and escape from the crustal sources and interact with the surrounding peridotite bodies, within a crust-mantle melange^11^, to form a veined mantle similar to by the composite mantle xenoliths reported in this study. Melting of such a metasomatized mantle may produce post-orogenic calc-alkaline to ultrapotassic magmas such as those occurring along the western Mediterranean^58^. SCLM sub continental lithospheric mantle.

Studies on ultra-high-pressure-temperature (UHTP) metamorphism indicate that rocks form the continental crust may be subducted to depths of 125–150 km reaching temperature above 1000 °C and undergo adiabatic melting during exhumation even in fluid-absent conditions^39^ (Fig. 6). Due to compositional heterogeneity of the crust, equilibrium melting is not easily achieved^40,41^, and whether a first melt produced has eutectic composition^42,43^ is matter of debate. Melts of rhyolitic composition might have been extracted during or after exhumation and have interacted with surrounding peridotite at lower temperature, as suggested by our MELTS model. Restitic metapelite xenoliths produced by melting of the lower crust^44^ have been found at Tallante^18,45^. These crustal xenoliths equilibrated with the peridotite at T-P conditions of 1090 °C and 0.7 GPa after melt extraction. This is consistent with the studied rhyolites being trapped in peridotite minerals closely resembling the minimum melt composition of the Qz-An-Or system at pressure conditions of 1 GPa^36–38^.

Textural, geochemical, and isotopic evidence of disequilibrium reported in this paper clearly indicates that metasomatism shortly preceded alkali-basalt volcanism, which is described as anorogenic^46–48^, although a recent study^49^ has demonstrated that these magmas partially interacted with of such a metasomatized mantle. So how can the anorogenic setting of Tallante magmatism be reconciled with the crustal subduction process identified by the HP rhyolite melts? The most plausible scenario is that previously subducted crustal material had been stored in the lithospheric mantle (or in a colder portion of the mantle wedge) and then heated during the rifting episodes, which triggered alkali basalt volcanism in this area. Subduction has been ongoing underneath the Betic Cordillera but basaltic magmatism and granitic metasomatism seem essentially coeval; thus, crustal mantle metasomatism may be activated when thermobaric conditions allowed for decompression melting of subducted continental lithosphere and mantle metasomatism. The buoyancy force of the subducted crust increases until slices/slivers of the continental crust detach from the down-going slab^50^, raised up and partially melt in nearly adiabatic condition^51^ (Fig. 6). The possibility that partially melted crustal blocks preserve and store a melt fraction during the exhumation of UHP rocks during slab-roll back has been recently demonstrated by numerical modeling^51^.

In this scenario, the rhyolite melt possibly segregates when the crustal lithologies end the retrograde P-T-t path and stabilize at the contact with relatively cold peridotite. Our data indicates that the interaction between H_2_O-undersaturated rhyolite and mantle peridotite occurred at temperature likely approaching 1090 °C, when massive orthopyroxene crystallization occurred (Supplementary Table 3). Due to their high reactivity and viscosity, the rhyolite melts cannot migrate far from their source, hence triggering the metasomatic reaction with the surrounding peridotite observed in the studied sample (Fig. 6). This process can take place at destructive plate margins, possibly in relation to slab roll-back. At the meso-scale, similar processes may operate where mantle peridotites are interlayered with migmatite terrains and crosscut by leucocratic veins, such as in Alpine-type massifs like Ronda and Beni Bousera^52^, in subduction-related settings, such as Himalaya^53^ and the Banda arc of Indonesia^54,55^.

Our study presents direct proof for the presence rhyolitic melts preserved within a veined lithospheric mantle, providing direct evidence of the interaction between crustal derived melts and supra-subduction lithospheric mantle. Rare occurrences of mantle-derived rhyolites in mantle settings have been reported, such as the rhyolite intrusion within the Krafla volcanic field (Iceland)^56^, and the rhyolite eruptions described at the northern East Pacific Rise MOR^57^, and in back-arc side of the Kuril arc^3^. However, based on chemical and isotopic analyses, these magmas are interpreted in terms of low-pressure partial melts of basaltic^56,57^ or low degree partial melting of metasomatized mantle^3^. Therefore, our findings differ from those mentioned above being crustal high silica melt in a peridotitic mantle. The main reason for this may be related to their genesis during isothermal decompression, resulting in melts with progressively higher H_2_O contents. Therefore, they increase their tendency of being highly reactive with the peridotite, which makes them ephemeral. In addition, the volumetric ratio between crustal rhyolite and peridotite tends to zero, as surrounding mantle may be approximated to an infinite reservoir.

The studied mantle xenoliths provide a key to explain the genesis of post-orogenic calc-alkaline to ultrapotassic magmatism occurring not only in the Betic region, but across the whole Western Mediterranean area^58^. These findings have even broader implications regarding the melting of mantle sources modified by subduction-related metasomatism. The geochemical and isotopic heterogeneity of the different portions of the studied xenoliths^21,22^ (i.e., between veins, reaction zones and surrounding peridotite, Figs. 3 and 5) argues against consolidated petrological principles that strictly infer identical isotopic composition between magmas and their mantle sources. Indeed, disequilibrium melting of such isotopically heterogenous veined mantle sources is strictly dependent on the mantle phases that prevalently contribute to the melting, and thus also by the melting degree.

## Methods

### Textural and chemical analyses

Samples were investigated using a field emission scanning electron microscopy (FE-SEM: ZEISS, model Merlin II), equipped with both energy (ED) and wavelength (WD) dispersive spectrometers. Thin sections of the sample were prepared and polished for textural and chemical analysis. Images of phases and their textures were first identified and collected by back-scattered electrons using a FE-SEM WD/ED probe. This kind of instrument has the capacity of focalize and maintain stable the beam current up to the high-resolution condition. Both the glass phase and the crystal phases were characterized; their chemical compositions were then accurately determined by combining ED/WD (equipped with five wavelength-dispersive spectrometers) spectrometers at the CERTEMA multidisciplinary laboratory of Grosseto (Italy). The operative conditions and standardizing procedures were opportunely selected in order to minimize the alkali loess and optimize the detection limits also in narrowing beam conditions, required from phases with a diameter <1 μm. The distribution of analytes between ED and WD spectrometer is consistent with both interfering peaks and synchronous measurement. Analytical conditions were 15 kV accelerating voltage and 2 nA beam current. The peak and total background counting times were set respectively at 10 s. In order to minimize the alkali-loss effect, different reference materials were used for reducing collected data from glass and minerals phases, respectively. In particular, rhyolite glass standard NMNH 72854 provided by Dept. of Mineral Sciences, Smithsonian Institution, was used for rhyolite glass analyses. The accuracy of analysis was checked by measurement of internal standards every 5 analyzed points whereas the probe current was monitored by Faraday Cup.

### In situ oxygen isotopes

In situ oxygen isotope analyses were performed using a CAMECA IMS 1280 HR2 ion microprobe at Centre de Recherches Pétrographiques et Géochimiques (CRPG, UMR 5873 CNRS-Université de Lorraine, Vandoeuvre les Nancy, France). The Cs+ primary ions beam of 2.5 nA were focused on a 10 µm diameter area and the electron gun used for the charge compensation. The negative secondary ions were measured with a mass resolution of 5000 (*M*/∆*M*) with an energy slit of 30 eV. Before each measurement, the sample was pre-sputtered for 90 s with a beam rastering on 15 µm to clean up the sample surface, then the secondary beam was automatically centred in the field aperture and contrast aperture and a beam rastering of 10 µm applied. The measurements are made on multicollection FC mode with counting time of 150 s. The instrumental mass fractionation was determined on the reference Bresil and Sonar 2. Bresil and Sonar 2 are two reference Quartz used two set up the instruments. The unknowns are normalized to Sonar 2, the external errors on the two sets of 5 and 7 measurements on Sonar 2 is 0.24‰, compared to 0.14‰ and 0.21‰ for each set of measurements. No time draft correction has been applied, as it does not significantly improve the precision of the measurements when the standards are not on the same holder than the samples. The internal errors on Standard and unknown range from 0.06 to 0.15‰. The secondary ion intensity relative to primary ion on samples is 80–100 % of the ones measured of standards, but there is no correlation between the secondary ion intensities and the δ^18^O, indicating a possible bias. The IMF correction is 6.35‰.

### Water content determination

Water contents of rhyolitic glasses was determined by using micro-Raman spectroscopy^59^. Raman spectra were acquired with a Horiba Lab Ram HR 800 spectrometer at the Department of Science, Roma Tre University. Data were collected using a 600 grooves/mm spectrometer grating and CCD detector. The estimation of the water content has been obtained via the following equation H_2_O (wt.%) = [WR(2700–4000)/SR(100–1500)]∙*m* where: (i) WR(2700–4000) is the area of water region from ~2700 to ~4000 cm^−1^; (ii) SR(100–1500) is the area of silicate region from ~100 to ~1500 cm ^−1^ and (iii) *m* is the linear fit coefficient from NBO/T equation of Bonechi et al. (2022). Error in water determination is estimated ±0.15 wt%.

## Supplementary information

Supplementary Information

## Data Availability

The data generated in this study (Supplementary Tables 1, 2, 4) and the MELTS simulation results (Supplementary Table 3) are provided in the Supplementary Information file.

## References

[1] Vielzeuf D, Holloway J (1988). Experimental determination of the fluid-absent melting relations in the pelitic system. Contrib. Mineral. Petrol..

[2] Brown M (1994). The generation, segregation, ascent and emplacement of granite magma: the migmatiteto-crustally-derived granite connection in thickened orogens. Earth-Sci. Rev..

[3] Takagi T, Orihashi Y, Naito K, Watanabe Y (1999). Petrology of a mantle-derived rhyolite, Hokkaido, Japan. Chem. Geol..

[4] Nichols GT, Wyllie PJ, Stern CR (1994). Subduction zone melting of pelagic sediments constrained by melting experiments. Nature.

[5] Hermann J, Spandler CJ (2008). Sediment melts at sub-arc depths: an experimental study. J. Petrol..

[6] Auzanneau E, Vielzeuf D, Schmidt MW (2006). Experimental evidence of decompression melting during exhumation of subducted continental crust. Contrib. Mineral. Petrol..

[7] Sekine WP (1982). The system granite-peridotite-H_2_O at 30 kbar, with applications to hybridization in subduction zone magmatism. Contrib. Mineral. Petrol..

[8] Sekine WP (1982). Phase relationships in the system KAISiO_4_–Mg_2_SiO_4_–SiO_2_–H_2_O as a model for hybridization between hydrous siliceous melts and peridotite. Contrib. Mineral. Petrol..

[9] Prouteau B, Scaillet B, Pichavant M, Maury R (2001). Evidence for mantle metasomatism by hydrous silicic melts derived from subducted oceanic crust. Nature.

[10] Mallik A, Nelson J, Dasgupta R (2015). Partial melting of fertile peridotite fluxed by hydrous rhyolitic melt at 2–3 GPa: implications for mantle wedge hybridization by sediment melt and generation of ultrapotassic magmas in convergent margins. Contrib. Mineral. Petrol..

[11] Foley S (1992). Petrological characterization of the source components of potassic magmas: geochemical and experimental constraints. Lithos.

[12] Codillo E, Le Roux AV, Marschall HR (2018). Arc-like magmas generated by mélange-peridotite interaction in the mantle wedge. Nat. Commun..

[13] Avanzinelli R, Lustrino M, Mattei M, Melluso L, Conticelli S (2009). Potassic and ultrapotassic magmatism in the circum-Tyrrhenian region: significance of carbonated pelitic vs. pelitic sediment recycling at destructive plate margins. Lithos.

[14] Conticelli S, Avanzinelli R, Ammannati E, Casalini M (2015). The role of carbon from recycled sediments in the origin of ultrapotassic igneous rocks in the Central Mediterranean. Lithos.

[15] Arai, S., Shimizu, Y. & Gervilla, F. Quartz diorite veins in a peridotite xenolith from Tallante, Spain: implications for reaction and survival of slab-derived SiO_2_- oversaturated melt in the upper mantle. *Proc. Japan Acad. Ser. B.* **79B**, 145–150 (2003).

[16] Beccaluva L, Bianchini G, Bonadiman C, Siena F, Vaccaro C (2004). Coexisting anorogenic and subduction-related metasomatism in mantle xenoliths from the Betic Cordillera (southern Spain). Lithos.

[17] Shimizu Y, Arai S, Morishita T, Yurimoto H, Gervilla F (2004). Petrochemical characteristics of felsic veins in mantle xenoliths from Tallante (SE Spain): an insight into activity of silicic melt within the mantle wedge. Trans. R. Soc. Edinb. Earth Sci..

[18] Bianchini G, Beccaluva L, Nowell GM, Pearson DG, Siena F (2011). Mantle xenoliths from Tallante (Betic Cordillera): Insights into the multi-stage evolution of the south Iberian lithosphere. Lithos.

[19] Platt JP, Behr WM, Johanesen K, Williams JR (2013). The Betic-Rif Arc and its orogenic hinterland: a review. Annu. Rev. Earth Planet. Sci. Lett..

[20] Duggen S, Hoernle K, van den Bogaard P, Garbe-Schönberg D (2005). Post-collisional transition from subduction- to intraplate type magmatism in the westernmost Mediterranean: evidence for continental-edge delamination of subcontinental lithosphere. J. Petrol..

[21] Dallai, L., Bianchini, G., Avanzinelli, R., Natali, C. & Conticelli, S. Heavy oxygen recycled into the lithospheric mantle. *Sci. Rep.* **9**, 8793 (2019). 10.1038/s41598-019-45031-3 PMC6584624 31217538

[22] Avanzinelli R (2020). S. Subduction-related hybridization of the lithospheric mantle revealed by trace element and Sr-NdPb isotopic data in composite xenoliths from Tallante (Betic Cordillera, Spain). Lithos.

[23] Sigmarsson O (2013). Formation of U-depleted rhyolite from a basanite at El Hierro, Canary Islands. Contrib. Miner. Pet..

[24] Shaw CSJ (2012). The effects of potassium addition on the rate of quartz dissolution in the CMAS and CAS systems. Contrib. Miner. Pet..

[25] Cesare B, Acosta-Vigil A, Bartoli O, Ferrero S (2015). What can we learn from melt inclusions in migmatites and granulites?. Lithos.

[26] Bartoli O, Acosta-Vigil A, Cesare B (2015). High-temperature metamorphism and crustal melting: Working with melt inclusions. Period. di Mineral..

[27] Dubinina E, Borisov A, Wiedenbeck M, Rocholl A (2000). SIMS oxygen isotope matrix effects in silicate glasses: quantifying the role of chemical composition. Chem. Geol..

[28] Stolper, E. & Epstein, S. *Stable Isotope Geochemistry: A Tribute to Samuel Epstein* (eds Jr. Taylor, H. P., O’Neil, J. R., Kaplan, I. R.) (The Geochemical Society, San Antonio, Texas, 1991).

[29] Baker JA (2000). Resolving crustal and mantle contributions to continental flood volcanism, Yemen: constraints from mineral oxygen isotope data. J. Petrol..

[30] Bindeman I (2008). Oxygen isotopes in mantle and crustal magmas as revealed by single crystal analysis. Rev. Mineral. Geochem..

[31] Mattey MDP, Lowry D, Macpherson CG (1994). Oxygen isotope composition of mantle peridotite. Earth Planet. Sci. Lett..

[32] Farver JR (2010). Oxygen and hydrogen diffusion in minerals. Rev. Mineral. Geochem..

[33] Matthews, A., Goldsmith, J., R. & Clayton, R. N. Oxygen isotope fractionations involving pyroxenes: the calibration of mineral-pair geothermometers. *Geochim. Cosmichim. Acta*, **47**, 631–644 (1983).

[34] Ghiorso MS, Sack RO (1995). Chemical mass transfer in magmatic processes IV. A revised and internally consistent thermodynamic model for the interpolation and extrapolation of liquid-solid equilibria in magmatic systems at elevated temperatures and pressures. Contrib. Mineral. Petrol..

[35] Asimow PD (1998). Algorithmic modifications extending MELTS to calculate subsolidus phase relations. Am. Mineral..

[36] Gualda GAR, Ghiorso MS, Lemons RV, Carley TL (2012). Rhyolite-MELTS: a modified calibration of MELTS optimized for silica-rich, fluid-bearing magmatic systems. J. Petrol..

[37] Gualda GA, R. Ghiorso MS (2013). Low-pressure origin of high-silica rhyolites and granites. J. Geol..

[38] Cesare B, Ferrero S, Salvioli-Marani E, Pedron D, Cavallo A (2009). “Nanogranite” and glassy inclusions: the anatectic melt in migmatites and granulites. Geology.

[39] Deng L-P, Liu Y-C, Gu X-F, Groppo C, Rolfo F (2018). Partial melting of ultrahigh-pressure metamorphic rocks at convergent continental margins: evidences, melt compositions and physical effects. Geosci. Front..

[40] Bea F (1996). Residence of REE, Y, Th and U in granites and crustal protoliths; implications for the chemistry of crustal melts. J. Petrol..

[41] Villaros A, Stevens GJ, Moyen F, Buick IS (2009). The trace element compositions of S-type granites: evidence for disequilibrium melting and accessory phase entrainment in the source. Contrib. Mineral. Petrol..

[42] Harris NBW, Ayres M, Massey J (1995). Geochemistry of granitic melts produced during the incongruent melting of muscovite: implications for the extraction of Himalayan leucogranite magmas. J. Geophys. Res..

[43] Acosta-Vigil A, London D, Morgan GB (2006). Experiments on the kinetics of partial melting of a leucogranite at 200 MPa H_2_O and 690–800 °C: compositional variability of melts during the onset of H_2_O saturated crustal anatexis. Contributions Mineral. Petrol..

[44] Vielzeuf D, Schmidt MW (2001). Melting relations in hydrous systems revisited: application to metapelites, metagreywackes and metabasalts. Contributions Mineral. Petrol..

[45] Bianchini G, Braga R, Langone A, Natali C, Tiepolo M (2015). Metasedimentary and igneous xenoliths from Tallante (Betic Cordillera, Spain): Inferences on crust–mantle interactions and clues for post-collisional volcanism magma sources. Lithos.

[46] Benito R (1999). Sr and O isotope constraints on source and crustal contamination in the high-K calc-alkaline and shoshonitic Neogene volcanic rocks of SE Spain. Lithos.

[47] Turner S (1999). Magmatism associated with orogenic collapse of the Betic–Alboran domain, SE Spain. J. Petrol..

[48] Zeck HP, Kristensen AB, Williams IS (1998). Post collisional volcanism in a sinking slab setting-crustal anatectic origin of pyroxene–andesite magma, Caldear Volcanic Group, Neogene Alboran volcanic province, southeastern Spain. Lithos.

[49] Casalini M (2022). Subduction-related lamproitic signature in intraplate-like volcanic rocks: the case study of the Tallante alkali basalts, Betic Chain, South-Eastern Spain. Ital. J. Geosci..

[50] Brun JP, Faccenna C (2008). Exhumation of high-pressure rocks driven by slab rollback. Earth Planet. Sci. Lett..

[51] Patino Douce, A. E. & McCarthy, T. C. *When Continents Collide: Geodynamics and Geochemistry of Ultrahigh-Pressure Rocks* (eds Hacker, B. R. & Liou, J. G.) (Kluwer Academic Publishers) 27–55 (1998).

[52] Gueydan F, Mazzotti S, Tiberi C, Cavin R, Villaseñor A (2019). Western Mediterranean subcontinental mantle emplacement by continental margin obduction. Tectonics.

[53] Burg J, Bodinier J, Chaudhry S, Hussain S, Dawood H (1998). Infra-arc mantle–crust transition and intraarc mantle diapirs in the Kohistan Complex (Pakistani Himalaya): petro-structural evidence. Terra Nova.

[54] Pownall JM, Hall R, Watkinson I (2013). Extreme extension across Seram and Ambon, eastern Indonesia: evidence for Banda slab rollback. Solid Earth Discuss..

[55] Pownall JM, Hall R, Armstrong RA (2017). Hot lherzolite exhumation, UHT migmatite formation, and acid volcanism driven by Miocene rollback of the Banda Arc, eastern Indonesia. Gondwana Res..

[56] Elders WA (2011). Origin of a rhyolite that intruded a geothermal well while drilling at the Krafla volcano, Iceland. Geology.

[57] Portner RA, Dreyer BM, Clague DA (2020). Mid-ocean-ridge rhyolite (MORR) eruptions on the East Pacific Rise lack the fizz to pop. Geology.

[58] Conticelli S (2009). Trace elements and Sr–Nd–Pb isotopes of K-rich, shoshonitic, and calc-alkaline magmatism of the Western Mediterranean Region: genesis of ultrapotassic to calc-alkaline magmatic associations in a post-collisional geodynamic setting. Lithos.

[59] Bonechi B (2022). Micro-Raman water calibration in ultrapotassic silicate glasses: application to phono-tephrites and K-foidites of Alban Hills. Chem. Geol..

[60] Blundy J, Cashman K (2001). Ascent-driven crystallisation of dacite magmas at Mount St Helens, 1980–1986. Contrib. Mineral. Petrol..

