# Supplementary material for: Quartz-bearing rhyolitic melts in the Earth’s mantle
Source: Nat Commun. 2022 Dec 15;13:7765. doi: 10.1038/s41467-022-35382-3 (PMC9755292; doi:10.1038/s41467-022-35382-3)
Supplement: Supplementary file 1 — Supplementary Information [file 41467_2022_35382_MOESM1_ESM.pdf]

Quartz-bearing rhyolitic melts in the Earth's mantle

Luigi Dallai, Gianluca Bianchini, Riccardo Avanzinelli, Etienne Deloule, Claudio Natali, Mario Gaeta, Andrea Cavallo, Sandro Conticelli

Supplementary Information

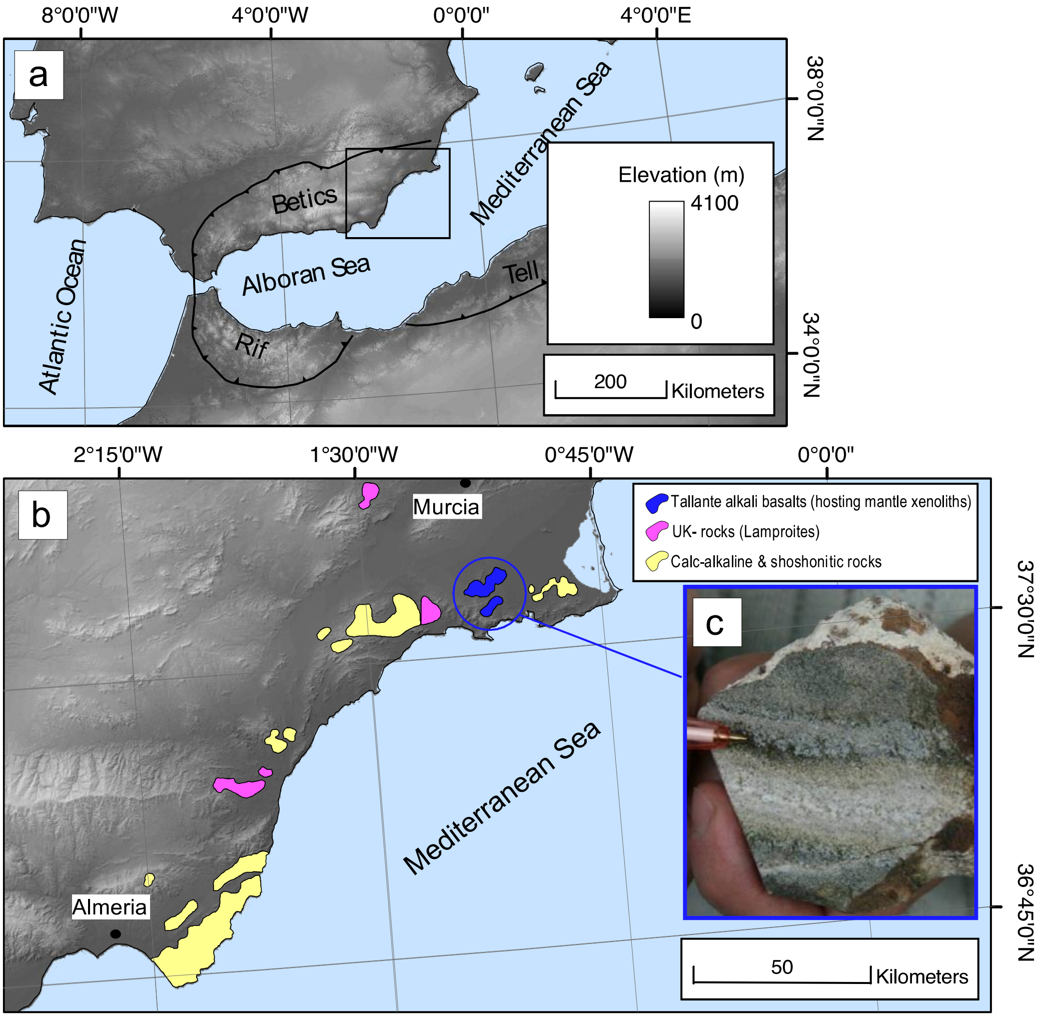

Supplementary Figure 1 – **Location and geodynamic setting**.
Topographic map of the Betic region with major tectonic features (a) and geographic distribution of Cenozoic magmatism (b). Composite (i.e., veined) mantle-derived ultramafic xenoliths (c) are hosted in the Tallante alkali basalts (2Ma) that represent the last magmatic episode in the region. Earlier subduction-related magmatism produced first calc-alkaline and shoshonitic products (12-8Ma) and then ultrapotassic rocks (i.e., lamproites; 10-6Ma). The Digital elevation model is a SRTM downloaded from https://srtm.csi.cgiar.org/srtmdata/.

Supplementary Table 1. Chemical compositions of glasses from the vein and the reaction zone of the composite xenolith TL112a and TL112b

	Type I -H_2O-undersaturated MI				Type II - Qz-bearing MI				Type III - Interstitial glasses			
**Analyses #**	**1 bis**	**2 bis**	**8bis**	**5bis**	**22**	**21a**	**21b**	**21c**	**4bis**	**15**	**14-1**	**14-2**
**SiO_2**	73.6	74.4	76.9	76.7	80.0	79.0	80.4	77.2	75.4	74.8	78.3	75.6
**TiO_2**	bdl	bdl	bdl	0.40	0.54	0.00	0.36	0.00	bdl	bdl	bdl	bdl
**Al_2O_3**	13.1	12.1	12.4	12.5	7.31	8.33	7.00	8.81	11.8	12.7	11.2	12.3
**FeO**	1.57	1.08	1.69	0.99	1.37	1.65	1.71	1.72	0.77	0.33	0.33	bdl
**MgO**	0.68	0.39	0.73	0.30	0.87	1.25	1.30	1.01	0.88	0.64	0.39	0.55
**CaO**	0.38	0.45	0.57	0.29	bdl	0.71	0.57	0.62	1.13	0.35	0.46	0.68
**Na_2O**	6.12	4.82	4.82	2.36	1.16	3.03	1.99	2.41	5.15	4.52	2.87	4.31
**K_2O**	4.35	4.11	3.99	4.34	3.36	2.94	2.55	2.69	2.38	3.56	2.88	3.29
**H_2O***	0.18	2.62	0.00	2.15	5.43	3.10	4.14	5.50	2.47	3.18	3.59	3.31
**H_2O****	1.00		0.00				5.73	10.38			3.68	
						**CIPW norm**						
Qz	25	30	30	46	62	47	57	51	34	34	50	37
Pl	44	40	41	22	10	27	20	25	47	41	28	41
Or	26	25	23	26	21	18	16	17	14	22	18	20
Crd	0	0	0	3	2	0	0	1	0	1	3	0
Di	2	2	2	0	0	3	0	0	3	0	0	0
Hy	4	2	4	2	4	5	6	6	2	2	2	1
Il	0	0	0	1	1	0	1	0	0	0	0	0

Legend: bdl = below detection limit; H_2O*: water content estimated by difference; H_2O**: water content estimated by micro-Raman.

Supplementary Table 2. In situ oxygen isotope analyses of the composite xenolith TL112

sample	spot	mineal phase	Zone	$d^{18}O$ ‰	s.e	description
TL112	TL112_Q1@1	quartz	Reaction Zone	8.28	0.14	Quartz crystal within Type II melt inclusion
TL112	TL112_Q1@2	quartz	Reaction Zone	9.30	0.16	Quartz crystal within Type II melt inclusion
TL112	TL112_Q1@3	quartz	Reaction Zone	8.58	0.15	Quartz crystal within Type II melt inclusion
TL112	TL112_Q1@4	quartz	Reaction Zone	9.07	0.15	Quartz crystal within Type II melt inclusion
TL112	TL112_Q1@5	quartz	Reaction Zone	9.49	0.16	Quartz crystal within Type II melt inclusion
TL112	TL112_S9-inc@1	quartz	Reaction Zone	9.00	0.15	Quartz crystal within Type II melt inclusion
TL112	TL112_S9-inc@2	quartz	Reaction Zone	8.45	0.14	Quartz crystal within Type II melt inclusion
TL112	TL112_S9-inc@3	quartz	Reaction Zone	8.05	0.14	Quartz crystal within Type II melt inclusion
TL112	TL112_S5@1	quartz	Vein	10.55	0.18	Anhedral qz crystal within type III glass in the siliceous vein
TL112	TL112_S5@2	quartz	Vein	11.07	0.19	Anhedral qz crystal within type III glass in the siliceous vein
TL112	TL112_S5@3	quartz	Vein	10.92	0.18	Anhedral qz crystal within type III glass in the siliceous vein
TL112	TL112_S5@4	quartz	Vein	12.14	0.21	Anhedral qz crystal within type III glass in the siliceous vein
TL112	TL112_S5@5	quartz	Vein	12.42	0.21	Anhedral qz crystal within type III glass in the siliceous vein
TL112	TL112_S5@6	quartz	Vein	12.47	0.21	Anhedral qz crystal within type III glass in the siliceous vein
TL112	TL112_S6@1	quartz	Vein	11.42	0.19	Anhedral qz crystal within type III glass in the siliceous vein
TL112	TL112_S6@2	quartz	Vein	11.93	0.20	Anhedral qz crystal within type III glass in the siliceous vein
TL112	TL112_S7@1	quartz	inner vein	13.43	0.23	Anhedral qz crystal within type III glass in the inner portion of the siliceous vein
TL112	TL112_S7@2	quartz	inner vein	13.97	0.24	Anhedral qz crystal within type III glass in the inner portion of the siliceous vein
TL112	TL112_S7@3	quartz	inner vein	13.56	0.23	Anhedral qz crystal within type III glass in the inner portion of the siliceous vein
TL112	TL112_S7@4	quartz	inner vein	14.14	0.24	Anhedral qz crystal within type III glass in the inner portion of the siliceous vein
TL112	TL112_S7@4	quartz	inner vein	14.14	0.24	Anhedral qz crystal within type III glass in the inner portion of the siliceous vein

Published data on mineral separates from the same sample (from Dallai et al 2019)

sample	mineal phase	Zone	$d^{18}O$ ‰	s.e	description	Source
TL112	olivine	host peridotite	5.73	0.13	mineral separate	Dallai et al., 2019, Scientific Reports
TL112	clinopyroxene	host peridotite	6.47	-	mineral separate	Dallai et al., 2019, Scientific Reports
TL112	clinopyroxene	host peridotite	6.21	0.03	mineral separate	Dallai et al., 2019, Scientific Reports
TL112	spinel	host peridotite	4.20	0.10	mineral separate	Dallai et al., 2019, Scientific Reports
						Dallai et al., 2019, Scientific Reports
TL112	orthopyroxene	Reaction zone	8.28	0.14	mineral separate	Dallai et al., 2019, Scientific Reports
						Dallai et al., 2019, Scientific Reports
TL112	orthopyroxene	Vein	9.84	0.05	mineral separate	Dallai et al., 2019, Scientific Reports
TL112	plagioclase	Vein	10.56	0.15	mineral separate	Dallai et al., 2019, Scientific Reports

Legend: 1 s.e. errors for SIMS measurements are based on the reprudicibility (1 sd) of the measurements of the Sonar 2 standard used for mass fractionation correction

Supplementary Table 3. MELTS simulation modelling the interaction between the rhyolitic melts and the peridotite mantle

	Input	
	P =1 GPa	
T(°C)	≥1130	890
	Metasomatic melt	Host peridotite
Sample	8bis	TL45
Mass	59	41
		Modal abundance
Ol		58
Opx		24
Cpx		11
Pl		5
Sp		2
Qz		
SiO_2	75.3	45.7
TiO_2	0	0.13
Al_2O_3	12.1	3.85
FeO*	1.66	7.78
MnO	0	0.12
MgO	0.72	38.9
CaO	0.56	3.23
Na_2O	4.72	0.29
K_2O	3.91	0.01
H_2O	1	0
Total	100	100

	Output									
	P =1 GPa									
T(°C)	1090		1070		970		890			
	Reagent phases	Crystallized phases	Reagent phases	Crystallized phases	Reagent phases	Crystallized phases	Reagent phases	Crystallized phases	Reagent phases	Crystallized phases
Ol	5.5		7.1		13.9		16.3		23.9	
Opx	1.5	11.9	2.9	16	5.7	31.4	8.3	40.8	9.9	52.1
Cpx	0.7		1.4		2.6		3.8		4.5	
Pl	0.3		0.6	6.6	1.2	26	1.7	37.6	2.1	33
Sp	0.1		0.2		0.5		0.7		0.8	
Qz						0.7		5.7		1.2
	Mineral proportions (%) are comulative									
	Melt									
	88.2		77.5		41.8		15.9		13.6	
SiO_2	74.8		75.6		78.3		78.3		77.9	
TiO_2	0		0		0		0		0	
Al_2O_3	12.7		12.3		10.4		9.65		10.4	
FeO*	0.88		0.66		0.17		0.14		0.12	
MnO	0		0		0		0		0	
MgO	0.97		0.83		0.41		0.42		0.44	
CaO	0.78		0.67		0.43		0.44		0.47	
Na_2O	4.86		4.6		3.42		3.37		3.1	
K_2O	4.01		4.29		5.04		3.32		3.28	
H_2O	1.03		1.13		1.82		4.33		4.32	
Total	100		100		100		100		100	

Calculated oxygen values	d^{18}O ‰ assumed	d^{18}O ‰ TL 45 lherzolite (Dallai et al 2019)
Ol		5.52
Opx		6.08
Cpx		6.03
Pl		6.32
Sp		4.35
Qz		
bulk	**14.14**	**5.73**

	d^{18}O ‰	d^{18}O ‰	d^{18}O ‰	d^{18}O ‰
Ol				
Opx	8.42	11.26	11.86	12.62
Cpx				
Pl		11.26	11.86	12.62
Sp				
Qz			11.86	12.62

	Typical Mantle values
Ol	5.19- 5.78 ‰
opx	5.68 - 6.47 ‰
cpx	5.03 - 6.62 ‰

The d^{18}O values of phases during the steps defined by thermodynamic model are calculated by mass balancing the O-isotope contribution of the peridotite and that from the rhyolitic melt. For mineral phases that are crystallised and reacted during the process (opx and pl), the reacting phases of each step have the isotope composition calculated in the previous step.

Supplementary Table 4. Micro-Raman analyses of melt inclusions.

		SR area	WR area	WR/SR	*m* coeff	H_2O wt.%
**Sample Tl112a**	Inclusion Type					
1bis	Type I	2864.19	3687.12	1.29	0.77	1.00
8bis	Type I	2437.64	0.00	0.00	0.77	0.00
**Sample Tl112b**						
21b	Type II	1955.46	14480.12	7.40	0.77	5.73
21c	Type II	2182.14	29281.36	13.42	0.77	10.38
14-1	Type III	2748.78	13070.96	4.76	0.77	3.68

Legend: SR: silicate region; WR: water region; *m* coefficient as function of NBO/T (Bonechi et al., 2022)

Supplementary References.

Bonechi B. et al. Micro-Raman water calibration in ultrapotassic silicate glasses: application to phono-tephrites and K-foidites of Alban Hills. Chemical Geology **597**, 120816 (2022)

Dallai, L., Bianchini, G., Avanzinelli, R., Natali, C. & Conticelli, S. Heavy oxygen recycled into the lithospheric mantle. Sci. Rep. **9** (2019).
